# Characterization and burden of Campania children health migration across Italian regions during years 2006–2010: chance and/or necessity?

**DOI:** 10.1186/1824-7288-38-58

**Published:** 2012-10-23

**Authors:** Pietro Vajro, Giulia Paolella, Egidio Celentano, Giuseppe Longo, Tullia Saccheri, Claudio Pinto, Giuseppe Masullo, Virginia Scafarto, Attilio Montano Bianchi

**Affiliations:** 1 Chair of Pediatrics, Medical School of the University of Salerno, Salerno, Italy; 2 ArSan, Regional Health Agency of Campania Region, Naples, Italy; 3 Department of Human Science, Philosophy and Education (DISUFF), University of Salerno, Salerno, Italy; 4 Department of Economics and Statistics Science, University of Salerno, Salerno, Italy; 5 University Hospital “San Giovanni di Dio e Ruggi d’Aragona”, Salerno, Italy

**Keywords:** Campania region, Southern Italy, Children, Health migration, Interregional mobility

## Abstract

**Background:**

To evaluate medical, economical and sociological variables underlying avoidable pediatric migration from Campania region.

**Methods:**

Analysis of years 2006–2010 hospital discharge records, extracted from the archive of Regional Health Agency (ArSan), classified by Major Diagnostic Categories, Aggregate Clinical Codes, Discipline of dismissal, Local Health Authorities of residence, and age group 0–14 years (excluding those of healthy newborns). Sociological variables were evaluated by questionnaires.

**Results:**

A total of 68,316 hospital discharge records were released by extra-regional structures. Major diagnostic categories and Discipline of dismissal indicated that the most implicated diseases (nervous system and mental disorders, hematology-oncology, and bone diseases) were not always of very high complexity. The total cost paid by the Campania Region was 124.700.000 Euros. The need for more specialized hospital pediatric units and/or with more pediatric subspecialties in the native region was pointed out by most of the self-administered questionnaires.

**Conclusions:**

Pediatric migration is an important phenomenon with evident implications. The identification of the most concerned sub-specialties here reported can give useful information aiming to assist in the improvement of the existing pediatric resources in Campania region in the wider context of the national global child health advancement.

## Introduction

Italian health system is based on regional economic resources, most of them being chronically deficient in Southern Italy. Each region has several Local Health Units (ASL), i.e. public authorities with legal personality, administrative, capital and management features, which provide health care organization in its own territory, and supply it through public or endorsed private structures. When a region is indebted, this determines a frequent reduction in adult and pediatric medical quality of care, even though the Italian health system aims to providing equality of the standards for all citizens [1].

One of the natural consequences is patients’ cross-border mobility [2-5]. This phenomenon however is not only Italian [6-17], and it has been correlated to important aspects not only in terms such as quality of services, and economic aspects, but also of waiting times, patient’s free choice of hospital, and doctor-patient relationships.

Studies in pediatric age are scarcely represented in international literature. The existing ones have shown that pediatric migration in search of better quality health care assistance is a relevant phenomenon particularly in Southern Italy regions, including Campania region [18]. Although presently Campania region may now rely on several qualified Regional Referral Centers for a number of Pediatric sub-specialties, mostly allocated in two Teaching Hospitals and in the Regional Children Hospital of Naples (Table 1), preliminary data suggested that Campania born children in several cases still have to migrate to obtain correct cures and care [5]. It is still doubtful if this is due to the chance and/or to real necessity [19]. 

**Table 1:** Major regional referral centers for pediatric sub-specialties in Campania region

**Regional referral center**	**Pediatric sub-specialties and some related supports**	
Federico II University Hospital-Naples		
	Pediatric home parenteral nutrition	
	Pediatric HIV infection	
	Pediatric diabetology	
	Inflammatory bowel disease (IBD) in pediatric age	
	Pediatric celiac disease	
	Diagnosis and treatment of cystic fibrosis	
	Pediatric rheumatological diseases	
	Rare diseases (coordinating center of 11 intra- regional units)	
	PKU	
	Clinical molecular biology, genetics and congenital metabolic disease diagnostic laboratory.	
SUN University Hospital-Naples		
	Neurofibromatosis 1	
	Pre-and post bone marrow transplantation	
	Pediatric diabetology	
	Pediatric thalassemia and other hemoglobinopathies	
	Pediatric celiac disease	
	Cardiac surgery and transplantation	
Children’s Hospital “Santobono Pausilipon- Annunziata”-Naples		
	Leishmaniasis	
	Pediatric Bone Marrow Transplantation	
	Organ transplantation	
	Retinopathy of prematurity	
	Hemoglobinopathies	
	Pediatric cochlear implants	
	PKU - Metabolic diseases and hypothyroidism screening	
	Pediatric Orthopedics	

The objective of our study is therefore to analyze with a multidisciplinary approach medical, sociological and economic conditions that underlie the phenomenon in the Campania Region, aiming to serve as a possible reference point for planning appropriate containment strategies valid for this, and possibly also for other indebted Italian regions with high pediatric migratory flows.

## Methods

The multidisciplinary research group consisted of the Chair of Pediatrics of the University of Salerno's School of Medicine and Surgery, the Departments of Sociology, and of Economics and Statistics of the University of Salerno, the “San Giovanni di Dio and Ruggi d’Aragona” Regional Teaching Hospital of Salerno, and ArSan (Regional Health Agency) of Campania Region.

Hospital admissions (years 2006–10) of patients aged 0–14 years -excluding those of healthy newborns- were analyzed through administrative hospital discharge records (SDO) available at the ArSan-Campania region database. SDO data were classified by MDC (Major Diagnostic Categories) that include 25 groups of organ diseases, ACC (Aggregate Clinical Codes) that comprise the ICD-9-CM (International Classification of Diseases, Ninth Revision) codes into a set of 259 small classes of homogeneous diagnoses and 231 types of intervention, Discipline of dismissal, ASL of residence, and age group 0–14 years.

The total adjusted cost was based on current rates (TUC) for hospital treatments.

Focus groups with pediatricians of regional provinces consisted in an interactive discussion about pediatric migration, and were structured to know their point of views about pediatric health care in Campania region. Sociological variables were evaluated through a self-administered questionnaire (Additional file 1) offered to a sample of parents of children migrated from Campania region and from Southern Italy admitted to the Pediatric Hospital Bambino Gesù of Rome, and to the Giannina Gaslini Institute of Genoa. Another set of questionnaires were administered directly by general pediatricians to parents of their patients known for a previous extra-regional migration to an Italian excellence hospital.

Finally, a survey of pediatric resources in Campania region was started by an University-Hospital-Pediatric Network coordinated by the Chair of Pediatrics of the University of Salerno.

## Results

**Figure 1:**
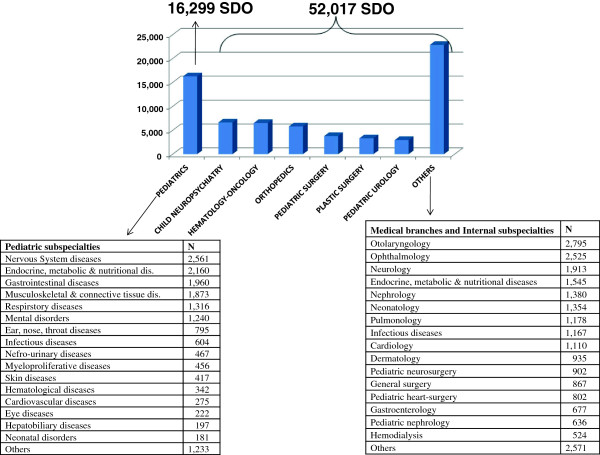
Most frequent causes of health migration in Campania region children.

The analysis of extra-regional pediatric SDO of years 2006–2010 showed that the total number of extra-regional Campania patients admissions was 68,316 (Figure 1), including 16,299 admissions at Units of Pediatrics, and 52,017 at Specialty Medical Centers (e.g. Stella Maris Foundation of Pisa specializing in neurological diseases, Italian Dermopatic Institute of Rome specializing in skin diseases, etc.). The column “others” includes several branches such as otolaryngology (n=2,795), ophthalmology (n=2,525), adult neurology (n=1,913), dermatology (n=935), pediatric neurosurgery (n=902), surgery (n=867), pediatric heart surgery (n=802), and also a number of pediatric clinical sub-specialties (n=32,370). A partial list of the most frequent sub-specialties (those with n SDOs ranging from 524 to 1,545 units) includes, in decreasing order, endocrinology, neonatology, pulmonology, infectious diseases, cardiology, gastroenterology, nephrology, hemodialysis.

The total adjusted cost paid by Campania Region for these health services amounted to 124.700.000 Euros.

**Figure 2:**
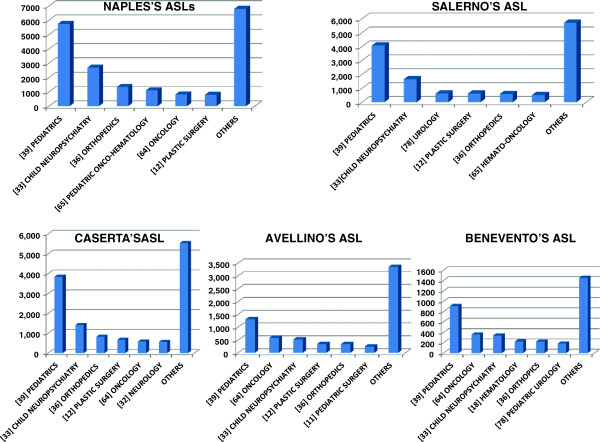
Most frequent causes of children migration subdivided for provinces.

The analysis of extra-regional admissions classified by ASL of residence showed a grossly comparable pattern of pediatric subspecialties migration, with the exception of an higher percentage of migration due to hematology-oncology diseases for Avellino and Benevento’s ASL residents, and to pediatric urology for Salerno’s ASL residents (Figure 2).

The Campania region escape index (i.e. the ratio between extra and intraregional hospitalizations) was 10.6%, with lesser values for the three Naples ASLs vs. the ASLs of the other four provinces (mean values 6.3% vs. 17.4%).

**Figure 3:**
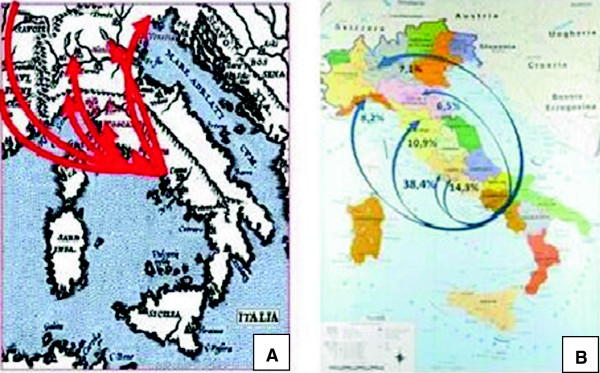
Pediatric migratory flows from Campania region in years 2002–06 (panel A) and 2006–10 (panel B).

Comparison of our data with those of a previous preliminary study on the same phenomenon during the years 2002–2006 (Andria et al, unpublished data) does not allow to find substantial differences in migratory flows, and confirms that the highest proportion of Campania children still migrates to Rome (in particular to the Pediatric Hospital Bambino Gesù) (Figure 3).

Table 2 shows the most prevalent diseases for each pediatric subspecialty classified by ACC. The overall analysis of ACC highlights that pediatric migration regards not only rare or complex diseases (i.e. inevitable mobility), but unfortunately also a large proportion of extra-regional hospitalizations for low complexity conditions.

**Table 2:** SDO (hospital discharge records) for the most prevalent diseases classified by Aggregate Clinical Codes (ACC)

**SDO Classified by Aggregate Clinical Codes**	**Total**
**Nervous System Disorders**	
[083] epilepsy and seizures	21.224
[084] headache, including migraine	4.112
[095] other nervous system disorders	3.356
**Mental Disorders**	
[654] developmental disorders	8.446
[655] childhood & adolescence disorders	2.176
[652] attention deficit/violent behavior disorders	1.243
**Gastrointestinal Diseases**	
[155] other gastrointestinal disorders	16.221
[251] abdominal pain	13.274
[135] intestinal infections	12.833
[142] appendicitis, appendix diseases	10.879
**Musculoskeletal System Diseases**	
[212] other bones/musculoskeletal diseases	3.774
[202] rheumatoid arthritis/related diseases	3.441
[204] other joint non-traumatic disorders	3.210
**Endocrine Nutritional/Metabolic Diseases**	
[058] other endocrine/nutritional metabolic disorders	3.367
[055] fluids and electrolytes disorders	2.297
[051] other endocrine disorders	2.211
**Respiratory Diseases**	
[124] acute and chronic tonsillitis	4.790
[122] pneumonia (not caused by STD/TBC)	3.497
[126] other infections of upper respiratory tract	3.315
**Renal and Urinary Tract Diseases**	
[215] congenital anomalies of genitourinary	13.099
[161] other kidney and ureteral diseases	10.023
[166] other male genital organs diseases	9.691
**Myeloproliferative Disorders**	
[045] chemotherapy and radiotherapy	8.702
[047] other unspecified benign tumors	5.352
[039] leukaemia	4.111

As shown in Table 2 and Figure 1, nervous system disease is the first cause of pediatric mobility, epilepsy, migraine, and mental retardation (ICD9, codes 317 and 319) being the major indications. Interestingly, during the study period the number of hospitalizations for pediatric mental disorders outside Campania region exceeded that of the intraregional ones (n= 6,624 vs. 5,317).

Eighty-two questionnaires were administered to parents of children coming from South Italy and admitted to extra-regional hospitals. Patients were hospitalized mainly in units specializing in neurology, gastroenterology, nephrology, cardiology and hematology-oncology disorders. Extra-regional hospitalization in most cases (35%) had been recommended by the pediatricians of the same southern Italy hospitals in which some patients were initially admitted, and only in a minority of cases by their own trusted/primary care pediatrician (18%) or by friends and relatives (16%). The majority of migrating families (65%) had received a clinical report regarding their child by the medical staff of their Southern Italy hospitals. In most cases subsequent follow-up had already been planned in the same extra-regional structure (73%). During hospitalization most caregivers slept at shelters (38%), or at the hospital itself (31%), or at hotels (23%).

**Figure 4:**
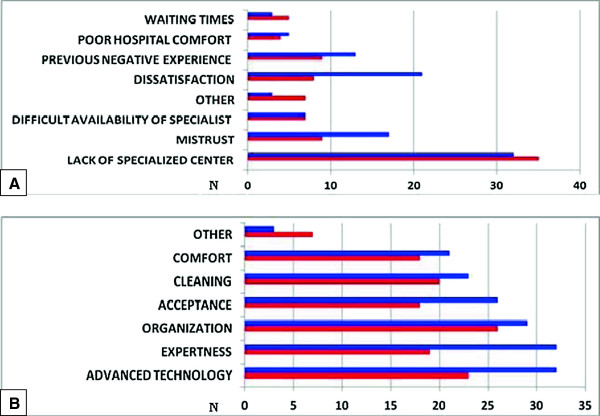
Results of self-administered questionnaires.

Most questionnaires reported a real or perceived lack of specialized centers in Southern Italy as the major reason for extra-regional admittances (81.5%). Advanced technology, organization and expertise were the most important differences detected by the interviews (Figure 4). The number of families with poor average education level was higher than those with secondary school or University education (58% vs. 42%).

Results of Campania region patients interviewed in Rome or Genoa and those of the set of questionnaires administered in Campania region by their primary care pediatricians did not show relevant differences as compared to those of the rest of Southern Italy patients.

Pediatricians’ experience gathered in focus groups and pediatric resources survey results confirmed that mental disorders are those especially felt to need unavoidable extra-regional hospitalizations, corresponding to our results shown above.

## Discussion and conclusions

This study analyzes medical and socioeconomic variables of the phenomenon of pediatric health mobility in Campania region. However we believe that the study methodology of our research may be used also for other Italian regions with high rates of patients migration.

In addition to causing stress to patients and their families, health migration determines significant costs to native region and subtracts economic resources to the development of their human and technological assets [20]. In general, international analyses in adults [6-17] have shown that perceived quality of services, waiting lists, and doctor’s expertness seem to represent important aspects for patients. Our families feel that pediatric extra-regional hospitalization in Campania region occurs mainly due to a (truly or perceived) lack of adequate and/or well organized facilities to provide updated assistance to children with complex diseases. However, unexpectedly, migration involved also conditions of medium or low complexity.

The analysis of our results underlines that some pediatric sub-specialty areas such as pediatric neurology, endocrinology, gastroenterology, and rheumatology are major causes of extra-regional admissions despite the existence of a number of qualified/certified Referral Regional Centers for the same pathologies.

A better insight into pediatric migratory flows brings to lights that more pronounced migratory flows of specific ASLs mirror the lack of local specialized spoke centers [e.g. hematology-oncology diseases in some provinces of Campania region (Avellino and Benevento) and for pediatric urology in Salerno’s province].

Looking at our data and at regional resources survey, it emerges that a concrete reinforcement and/or rationalization of some existing pediatric resources (e.g. musculoskeletal and connective tissue, urology, child neuropsychiatry) might contribute to reduce the proportion of avoidable migration. For others (e.g. gastroenterology, nephrology, hematology-oncology, diabetology) it is possible that some existing excellences are not adequately publicized or efficiently interfaced with patients and/or their trusted pediatricians and peripheral general hospitals. A more complete survey of these and other pediatric resources is therefore needed since it might be useful to better acknowledge efficient units and define consequently the “avoidable” and “unavoidable” components of regional pediatric health mobility. In this regard one must consider the relevant role of family physicians in our country’s health care system: they not only act as a first point of consultation for patients, but also determines whether or not to refer them to subspecialty care or to other centers. It is possible that, at least for some cases, no sufficient communication between physicians at the Campania hospitals and primary care pediatricians still exists. It will therefore be worthwhile to check if extension -also to other provinces- of regional pediatric resources surveys as the one started by our University-Hospital-Pediatricians Network in Salerno, might have a positive effect on primary care pediatricians’ decision-making processes.

It must also be verified whether 1. novel joint interregional ventures such as that for pediatric hematology-oncology between Regional Children’s Hospital Santobono-Pausilipon, Naples and Giannina Gaslini Institute, Genoa, 2. the recent experiences of delocalized general or specializing sections of Pediatric Hospital Bambino Gesù in Molise, Calabria, Basilicata, Sicily and Campania itself, and 3. the institution of the teaching hospital in the Salerno’s area, overall will contribute to cut costs for pediatric patients healthcare and to improve local care/cure qualities. Similarly, one must verify if the present tentative of unifying costly duplicate pediatric divisions existing in small hospitals that often insist on the same restricted geographical areas, may be the right cure for cutting costs. This might help in raising funds to obtain centralized adequate technological and staff updating at teaching hospital level.

In any cases, a regional multidisciplinary observatory of pediatric migration finally might be useful to evaluate prospectively the effectiveness of adopted corrective measures.

## Abbreviations

ACC: Aggregate Clinical Codes; ArSan: Regional Health Agency; ASL: Local Health Units; ICD-9-CM: International Classification of Diseases, Ninth Revision; MDC: Major Diagnostic Categories; SDO: Hospital discharge records; TUC: Current rates for hospital treatments.

## Competing interests

The authors declare that they have no competing interests.

## Authors' contributions

PV supervised the study and the final MS draft; GP prepared bibliographical background, elaborated SDO data, and organized Focus groups and Questionnaires; EC and GL extracted and commented on SDO data from the regional archive of the ArSan; TS and GM participated in the sociological analysis; CP participated in the economical analysis; VS and AMB participated in the organization of *ad hoc* pediatric meetings. All authors read and approved the final manuscript.

## Funding

This work was partially supported by University of Salerno FARB (ex MURST 60%) year 2011.

## Supplementary Material

Additional file 1 **Appendix 1.** Multicenter epidemiological investigation on pediatric mobility -spring 2012. Click here for file
