# Supplementary material for: Characterization and burden of Campania children health migration across Italian regions during years 2006–2010: chance and/or necessity?
Source: Ital J Pediatr. 2012 Oct 23;38:58. doi: 10.1186/1824-7288-38-58 (PMC3514259; doi:10.1186/1824-7288-38-58)
Supplement: Additional file 1 — Appendix 1. Multicenter epidemiological investigation on pediatric mobility -spring 2012. [file 1824-7288-38-58-S1.docx]

**Appendix 1**

**MULTICENTER EPIDEMIOLOGICAL INVESTIGATION ON PEDIATRIC MOBILITY -SPRING 2012**

Dear Madam, Dear Sir,

In order to better understand the reasons that led you to address to a center of excellence outside your region, please fill this questionnaire prepared by the Chair of Pediatrics, Faculty of Medicine, University of Salerno.

Data obtained will be used - anonymously and not identifiably– for a research of the University of Salerno, funded by MIUR.

Prof. Pietro Vajro – School of Medicine, University of Salerno - tel. 089.672409; 965 016

CENTER OF ADMISSION: _______________________________________________________________

PATIENT GENDER: ☐ M ☐ F ; AGE: __ mos. ___year Place of Birth ____________

               Work, Last school / University attended

FATHER: ______________________________________

MOTHER:______________________________________

NUMBER OF PEOPLE COMPOSING YOUR FAMILY: ____

RESIDENCE:

Region: ____________ Province: ___________ City/ Country: _________________

1. What is the disease that affects your son/daughter?

_________________________________________________________________________

2. Did you receive a (suspected) diagnosis before this admission? ☐ YES ☐ NO

3. How much time has passed since the beginning of the disease? ____ Years ___ months

4. Have you made a previous hospitalization in your region? ☐ YES ☐ NO,

       - If YES, did you have any problem that prompted you to move? ☐ YES ☐ NO,

       - If YES, can you list the problems? _________________________________________________________

5. Did you make other admissions at this hospital for the current pathology? ☐ YES ☐ NO

- If YES, how much time has passed since the first hospitalization? ________________________

6. Have you already made other admissions outside your region due to health problems? ☐ YES ☐ NO

- If YES, at what centers? ____________________________________________

7. Did you previously consult specialists in your region? ☐ YES ☐ NO

8. Have you been recommended for this admission by your pediatrician? ☐ YES ☐ NO,

- If NO, who had suggested this admission? ☐FRIENDS ☐RELATIVES ☐HOSPITAL ☐ OTHER

     Specify: ________________________________________________________

9. If the treatment has been recommended by pediatricians or an hospital, did you receive a clinical report?

☐ YES ☐ NO

10. Did you choose spontaneously the admission to this hospital? ☐ YES ☐ NO

- If YES, which of these reasons did you mostly influence?

☐ a. Distrust in your origin region’s hospital

☐ b. Lack of a specialized center for the suspected disease

☐ c. Previous negative experience in your region

☐ d. Dissatisfaction for the proposed treatment by regional specialists and/or structures

☐ e. Waiting list in your regional structures

☐ f. Lack of available specialists in your region

☐ g. Poor comfort in your regional pediatric structures

- Other: ______________________________________________________

11. Which are the differences that you have seen between this hospital and those in your region?

☐ a. Cleaning

☐ b. Comfort

☐ c. Organization

☐ d. Advanced technology

☐ e. Acceptance

☐ f. Competence

☐ g. Other

Please specify: _________________________________________________

12. Do you think that the structures in your region are more disorganized and/or poorly equipped? ☐ YES ☐ NO

13. Do you have relatives or friends in this city? ☐ YES ☐ NO

14. Where have you been lodging during the period of hospitalization?

☐HOSPITAL ☐HOTEL ☐ APARTMENTS AND/OR SHELTERS ☐ RELATIVES OR FRIENDS

15. Did you receive a program for the follow-up? ☐ YES ☐ NO

-If YES, where will it be performed? ☐ at your origin region’s hospital ☐ at this hospital
